# Tuberous sclerosis complex diagnosed from oral lesions

**DOI:** 10.1590/1516-3180.2013.1315441

**Published:** 2013-10-01

**Authors:** Leonardo de Jesus Araújo, Guilherme Braga Muniz, Edmilson Santos, João Paulo Versiani Ladeia, Hercílio Martelli, Paulo Rogério Ferreti Bonan

**Affiliations:** I MSc. Researcher, Postgraduate Program on Health Sciences, Universidade Estadual de Montes Claros (Unimontes), Montes Claros, Minas Gerais, Brazil.; II Medical Student. Scientific Initiation Program, Institute of Health Sciences, Faculdades Unidas do Norte de Minas (Funorte), Montes Claros, Minas Gerais, Brazil.; III MD. Full Professor, Scientific Initiation Program, School of Medicine, Institute of Health Sciences, Faculdades Unidas do Norte de Minas (Funorte), Montes Claros, Minas Gerais, Brazil.; IV MBA. Researcher, Institute of Exact and Biological Sciences, Universidade Federal de Ouro Preto (UFOP), Ouro Preto, Minas Gerais, Brazil.; V PhD. Full Professor, Postgraduate Program on Health Sciences, Universidade Estadual de Montes Claros (Unimontes), Montes Claros, Minas Gerais, Brazil.

**Keywords:** Diagnosis, Tuberous sclerosis, Neurocutaneous syndromes, Pathology, Phenotype, Diagnóstico, Esclerose tuberosa, Síndromes neurocutâneas, Patologia, Fenótipo

## Abstract

**CONTEXT:**

Tuberous sclerosis complex (TSC) is a genetic disease in the group known as neurocutaneous syndromes, with dominant autosomal inheritance. It is characterized by skin and adnexal lesions and central and peripheral nervous system tumors, with neurological and psychiatric findings. It may affect the heart, kidneys, eyes, face, bones, lungs, stomach and dentition.

**CASE REPORT:**

We present the case of a 66-year-old man with dermatological signs that included hypopigmented maculae, confetti-like lesions, shagreen plaque, angiofibromas on nasolabial folds, neck and back, nail dystrophy and periungual fibromas on fingers and toes. An electroencephalogram produced normal results, but magnetic resonance imaging showed a nodular image measuring 1.2 x 1.0 cm close to the Monro foramen, which was similar to cerebral parenchyma and compatible with a subependymal giant-cell astrocytoma. A conservative approach was taken, through control imaging examinations on the lesion for seven years, with absence of any expansive process or neurological symptoms. Abdominal ultrasonography revealed a solid, heterogenic and echogenic mass with a calcified focus, measuring 4.6 x 3.4 cm, in the rightkidney, compatible with angiomyolipoma. The patient was treated by means of complete nephrectomy because of malignant areas seen on histopathological examination and died one month after the procedure. This case report illustrates the importance of oral clinical findings such as dental enamel pits and angiofibromas in making an early diagnosis of TSC, with subsequent screening examinations, treatment and genetic counseling.

## Abstract (pt)

**CONTEXTO:**

O complexo esclerose tuberosa é uma doença genética pertencente ao grupo das facomatoses, de herança autossômica dominante, caracterizada por lesões acometendo pele e anexos, além de tumores do sistema nervoso central e periférico, com presença de achados neurólogicos e psiquiátricos, podendo acometer coração, rins, olhos, dentes, mucosa oral e outros órgãos.

**RELATO DE CASO:**

Apresentamos o caso de um paciente do sexo masculino, 66 anos de idade, que apresentava diversos sinais dermatol ógicos, como máculas hipopigmentadas, lesões em confete, placas tipo *shagreen*, angiofibromas nas regiões cervical, nasolabial e dorsal, distrofias ungueais e fibromas nos dedos das mãos e pés. Embora o encefalograma não tenha mostrado alterações, a ressonância nuclear magnética apresentou imagem nodular com aspecto semelhante ao parênquima cerebral, medindo 1.2 x 1.0 cm e próxima ao forame de Monro, compatível com astrocitoma subependimário de células gigantes. Abordagem conservadora foi escolhida por meio de controle imaginológico da lesão por sete anos, com ausência de sintomas neurológicos ou processos expansivos. A ultrassonografia de abdômen total revelou massa medindo 4.6 x 3.4 cm, com áreas ecogênicas e heterogêneas, apresentando focos de calcificação no rim direito, com padrão semelhante ao de um angiomiolipoma. O paciente foi submetido a nefrectomia total, devido à presença de áreas de malignidade ao exame histopatológico, e evoluiu para óbito um mês após o procedimento. Este relato de caso ilustra a importância de achados clínicos orais, tais como lesões no esmalte dentário e angiofibromas, para o diagnóstico precoce dessa doença e posterior rastreamento, tratamento e aconselhamento genético.

## INTRODUCTION

Tuberous sclerosis complex (TSC) or Bourneville's disease, is a neurocutaneous syndrome (registered in the Online Mendelian Inheritance in Man as MIM 191100) characterized by autosomal dominant inheritance, with high prevalence of de novo mutations and variable expression.^(^ ^1^ ^-^ ^3^ ^)^ Among the reported findings, lesions of the skin, adnexa, central nervous system, heart, kidneys and other organs stand out.^(^ ^1^ ^-^ ^4^ ^)^ The prevalence is estimated to be 1:6000 live births,^(^ ^2^ ^,^ ^5^ ^)^ and two thirds of all cases do not have any familial history.^(^ ^2^ ^,^ ^4^ ^)^ The phenotypic expression of TSC is highly variable and, in some cases, it may be difficult to establish a definitive clinical diagnosis.^(^ ^3^ ^)^ Generally, the diagnosis is made based on multiple clinical criteria that are categorized into major and minor features. At the Tuberous Sclerosis Consensus Conference of 1998, the clinical diagnostic criteria of TSC were revised and a new classification system based on major and minor findings was established.^(^ ^6^ ^)^ The presence of two major features, or one major and two minor features, is sufficient for a definitive diagnosis.^(^ ^1^ ^-^ ^4^ ^)^ Over recent years, mutation analysis has become an additional diagnostic tool in cases of familial as well as sporadic TSC.^(^ ^7^ ^)^

Two disease-determining genes have been defined, named TSC1 and TSC2, on chromosomes 9q34 and 16p13, respectively. The TSC1 gene has 23 exons and its protein product is hamartin. The TSC2 gene has 41 exons in the coding region and a leader exon with a transcript that exists as multiple isoforms; its protein product is tuberin. Both hamartin and tuberin are tumor-suppressor proteins that are involved in cell growth and differentiation.^(^ ^7^ ^)^

Considering the rarity of diagnosing TSC based on oral lesions, the aim of this paper was to present a case report with emphasis on the importance of identifying of dental enamel pits and oral angiofibromas for diagnosing this disease. 

## CASE REPORT

**Figure 1:**
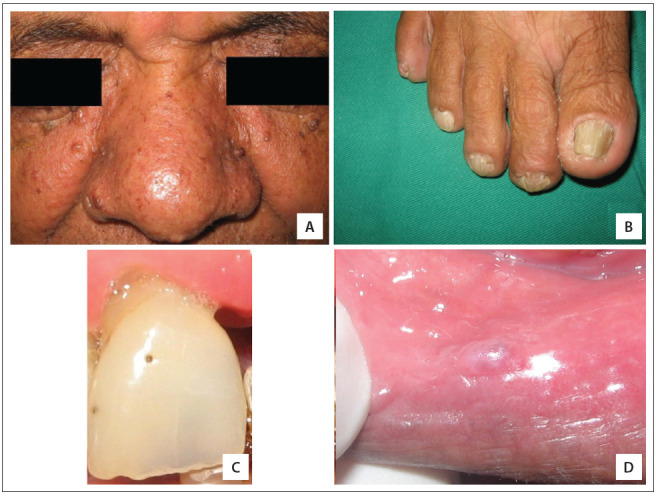
Clinical features identified in the patient affected by tuberous sclerosis complex. (A) Multiple facial papules affecting the nasolabial folds and the nose, compatible with angiofibromas; (B) non-traumatic ungual and periungual fibromas on the right foot; (C) enamel pitting in the permanent upper central incisor tooth; and (D) fibrous proliferation in the lower-lip mucosa, without apparent traumatic factors (angiofibroma).

**Figure 2:**
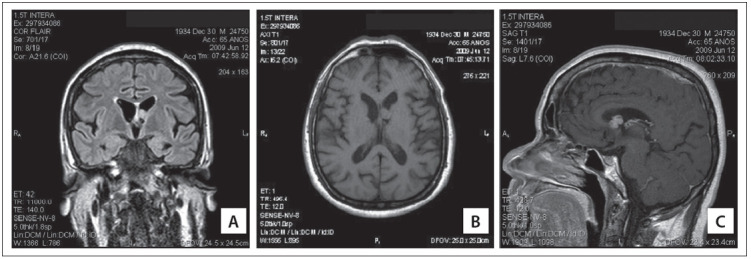
Neurological tuberous sclerosis complex features detected by means of magnetic resonance imaging (MRI) in a 1.5-T scanner. (A) Coronal and (B) axial FLAIR (fluid acquisition inversion recovery) magnetic resonance imaging (MRI), demonstrating the Monroforamen nodule, compatible with subependymal giant-cell astrocytoma, with and without administration of gadolinium contrast (GdDTPA), respectively; (C) sagittal T1 MRI after GdDTPA, showing a hyperintense signal from the tumor.

A 66-year-old non-Caucasian man was referred to the Stomatology Clinic of the State University of Montes Claros (Universidade Estadual de Montes Claros, Unimontes), state of Minas Gerais, Brazil, due to the presence of fibrous prolifera-tive lesions in the oral mucosa. Intraoral examination revealed hypoplastic defects on the vestibular face of the upper central incisor and canines and fibrous proliferations in the lowerlip mucosa, without apparent traumatic factors. The patient reported that he had been a tobacco user since his teenage years (Figure 1). The labial lesion was excised, and microscopic analysis using hematoxylin and eosin staining revealed a lesion compatible with oral angiofibroma (Figure 2). The association of dental enamel pits and gingival angiofibromas led to the clinical hypothesis of TSC and a search for skin lesions was performed.

The patient's medical history included intestinal ulcers and hematemesis, which had been treated using omeprazole. There were positive reports of convulsive crises, with the last episode in 1999, which had been controlled using phenobarbital. The family's history included occurrences of hypertension, diabetes mellitus, cancer and TSC. 

General clinical examination showed hypopigmented maculae, confetti-like lesions, shagreen plaque, angiofibromas on nasolabial folds, neck and back, nail dystrophy and periungual fibromas on hands and feet (Figure 1), seen under Wood's light. On physical examination, the patient did not show any signs or symptoms of cardiovascular, endocrine, respiratory, immune or musculoskeletal disorders. 

Brain magnetic resonance imaging was performed in a 1.5-T scanner including T1, T2, and FLAIR (fluid acquisition inversion recovery), and showed a nodular image similar to cerebral parenchyma, measuring 1.2 x 1.0 cm, with projection of the anterior cornu of the left lateral ventriculum, close to the Monro foramen (Figure 2). A conservative approach was chosen for this case, with control imaging examinations over a seven-year period, without any expansive process. These findings were compatible with a subependymal giant-cell astrocytoma. An electroencephalogram produced normal results and no evidence of signs or symptoms of psychopathological processes.

Abdominal ultrasonography was also requested. Although this showed that the viscera were normal, there was a solid, heterogenic and echogenic mass with a calcified focus inside, measuring 4.6 x 3.4 cm, in the right kidney, which was compatible with angiomyolipoma. Kidney function was normal and asymptomatic, and urine analyses did not show proteinuria. Echocardiography, additional routine laboratory tests and fundoscopy examinations were normal. 

The patient was admitted to the Santa Casa Hospital of Montes Claros, Minas Gerais, Brazil, for laparoscopic examination and biopsies on his right kidney. Histopathological examination on the material obtained was compatible with angiomyolipoma, but also revealed important areas of malignancy consistent with renal cell carcinoma. The patient then underwent medial longitudinal laparotomy and complete nephrectomy of right kidney. He died one month after the procedure. Permission to perform an autopsy was not granted. 

Prior approval to report on this case was obtained from the Institutional Ethics Committee (protocol no. 1047/08) and the patient signed an informed consent statement. We followed the evaluation and management recommendations put forward by Roach et al.^(^ ^6^ ^)^ This case was compatible with the diagnostic criteria established for TSC, which were confirmed by the clinical, imaging and histopathological findings and by the presence of a family history. 

## DISCUSSION

TSC is one of a group of related disorders known as neurocutaneous syndromes or phakomatosis.^(2),(3),(8)^ Other than TSC, the other major disorders in this group include types 1 and 2 neurofibromatosis, Sturge-Weber syndrome, ataxia-telangiectasia and von Hippel-Lindau disease.^(2),(3),(8)^ The term TSC refers to multiple sclerotic masses scattered throughout the cerebrum. TSC was first described by von Recklinghausen in 1862 and in more recent times was reported by Bourneville, Pringle and Vogt.^(^ ^9^ ^-^ ^11^ ^)^ The most common features of TSC include facial angiofibromas, hypopigmented cutaneous maculae, shagreen patches in the lumbar area, cerebral cortical tubers, subependymal nodules, subependymal giant-cell astrocytomas, cardiac rhabdomyomas and renal angiomyolipomas. Its minor features may include hamartomatous rectal polys, non-renal hamartomas and multiple renal and bone cysts.^(2-4),(6)^

Our patient presented classical cutaneous findings, including multiple angiofibromas on his face. These hamartomatous lesions are preferentially located on the nasolabial folds, malar regions (where they usually present symmetrically, in the form of "butterfly wings") and the nose and chin.^(2-4),(6),(12)^ The differential diagnosis includes acne vulgaris and dermatosis papulosa nigra^(3),(13),(14)^ (Figures 1 and 2). Several cosmetic treatments have been described including curettage, surgical excision, cryosurgery, electrosurgery, dermabrasion, pulsed dye laser and CO2 laser. However, the recurrence rates are considered to be high.^(2),(13),(15)^

Central nervous system involvement in TSC invariably presents with cortical tubers, subependymal glial nodules, white matter hamartomas and subependymal giant-cell astrocytoma. Neurological manifestations range from slight or even nonexistent to extremely severe symptoms. The most common neurological finding is seizures. However, other manifestations such as mental retardation of differing degrees and obstructive hydrocephaly secondary to tumor growth are frequently seen. One of the most important complications in patients with TSC is the development of subependymal giant-cell astrocytomas close to the Monro foramen. Imaging techniques, especially brain magnetic resonance, are now well established for diagnosing and following up TSC patients.^(3),(16)^

Kidney involvement frequently occurs in TSC cases. The main manifestations are angiomyolipomas, cysts and renal malignancies.^(2-4),(6)^ Renal angiomyolipoma is a benign tumor composed of three different types of tissue: fatty, smooth muscle and vascular.^(^ ^17^ ^)^ Angiomyolipoma often occurs in association with tuberous sclerosis between the second and third decades of life in isolated form.^(^ ^17^ ^,^ ^18^ ^)^ It primarily affects women between the fourth and seventh decade of life. The most common signs and symptoms are abdominal pain, palpable abdominal mass, hematuria and other consequences of intra-tumoral hemorrhage.^(17)(,19)^ The latter occurs in approximate 25% of the patients, and 10% of these patients may present hypovolemic shock in the acute phase. The symptoms and complications of angiomyolipoma are related to its size and rapidity of growth. Lesions greater than 4 cm indicate a greater risk of complications, such as hemorrhage. According to the literature, the treatment depends on the lesion size.17 Between 40% and 80% of individuals with TSC present renal angiomyolipoma with multiple bilateral asymptomatic tumors, which may be associated with cysts and, less frequently, with renal carcinoma.^(17),(18),(20)^ Total nephrectomy should be used very rarely; it is only justified in cases of uncontrollable bleeding that is a risk to the patient's life, or in cases of central tumors, extensive necrosis, inflammation of the renal tissue or presence of renal carcinoma in the same kidney, as in the present case.^(^ ^17^ ^)^

Oral manifestations of TSC are quite frequent and are characterized mainly by fibrous hyperplasia (angiofibromas) and dental enamel pitting.^(2),(15)^ Angiofibromas are frequently located in the anterior portions of the gingiva, but they are not rare on the lips, tongue, and palate. In addition to TSC, other syndromes may include facial and oral papules/nodules in their clinical spectrum, including Cowden syndrome, Birt-Hogg-Dubé syndrome and multiple endocrine neoplasia type 1.^(2,3),(21)^ Dental enamel pitting is observed in up to 100% of the patients with TSC. Dental pits can also be observed in the general population, but at lower frequency and with fewer lesions than in TSC cases. Enamel pits are also observed in cases of pitted hypoplastic amelogenesis imperfecta, vitamin D-dependent rickets, pseudohypoparathyroidism and junctional epidermolysis bullosa.^(2),(22)^

A systematic survey of indexed articles in the Medline/PubMed, Embase, Lilacs, Scirus, SciELO and Cochrane Library databases revealed that, to date, few articles have emphasized the importance of oral lesions such as concomitant dental enamel pits and oral angiofibromas in diagnosing this disease. The papers surveyed are described in Table 1. 

**Table 1:** Results from systematic search of indexed articles in medical databases performed on April 9, 2012, using descriptors for the main clinical findings observed in this case report*

Database*	Search strategy	Results
PubMed	(("Tuberous Sclerosis"[MeSH]) OR (Tuberous Sclerosis)) AND (("Dental Enamel" [MeSH]) OR (Dental Enamel) OR (Dental Enamel Pits) OR ("Angiofibroma" [MeSH]) OR Angiofibroma)	179
Lilacs or SciELO	((Tuberous Sclerosis) OR (Esclerose Tuberosa) OR (Esclerose Tuberosa) OR (Esclerosis Tuberosa) OR (Doença de Bourneville Epiloia) OR (Facomatose de Bourneville) AND ((Dental Enamel) OR (Dental Enamel Pits) OR (Esmalte Dentário) OR (Esmalte Dental) OR (Cutícula Dentária Esmalte) OR (Cutícula de Esmalte) OR Angiofibroma)	3
Embase	((Tuberous Sclerosis )) AND ((Dental Enamel) OR (Dental Enamel Pits) OR Angiofibroma)	62
Scirus	((Tuberous Sclerosis)) AND ((Dental Enamel) OR (Dental Enamel Pits) OR Angiofibroma)	610

* Using the same search strategy in the Cochrane Library databases, only one result was found on the same date

## CONCLUSION

This report on a case of TSC illustrates that proper identification of clinical oral features such as dental enamel pits and angiofibromas may help towards achieving early diagnosis of this disease and, ultimately, towards starting appropriate screening examinations, treatment and genetic counseling. 
